# Sustained metabolic reduction and hypothermia in humans

**DOI:** 10.1371/journal.pone.0321117

**Published:** 2025-04-21

**Authors:** Katharyn L. Flickinger, Alexandra J. Weissman, Francis X. Guyette, Ryann DeMaio, Andrea Jonsson, Victor Wu, Jenna L. Monteleone, Emma A. Zurowski, Jonathan Birabaharan, Daniel J. Buysse, Philip E. Empey, Thomas D. Nolin, Raymond E. West III, Clifton W. Callaway

**Affiliations:** 1 Department of Emergency Medicine, University of Pittsburgh School of Medicine, Pittsburgh, Pennsylvania, United States of America; 2 University of Pittsburgh School of Pharmacy, Pittsburgh, Pennsylvania, United States of America; 3 Department of Psychiatry, University of Pittsburgh School of Medicine, Pittsburgh, Pennsylvania, United States of America; University of Veterinary Medicine Vienna: Veterinarmedizinische Universitat Wien, Austria

## Abstract

Metabolic reduction is an adaptation employed by animals encountering environmental stressors or scarce resources. Lowering metabolism in humans may be useful to reduce consumables, oxygen utilization and carbon dioxide excretion. This is relevant for payload optimization or resource-restricted scenarios such as long-duration spaceflight or austere terrestrial environments (e.g., Arctic/Antarctic, submarine, cave or mine extraction). We previously demonstrated intravenous and single oral or sublingual doses of dexmedetomidine reduce oxygen consumption, wakefulness, and core body temperature in healthy humans. However, longer-acting dosing strategies are required to achieve greater levels of metabolic reduction. We explored whether a sublingual loading dose followed by subcutaneous infusion (SQI) of dexmedetomidine with and without surface cooling can decrease metabolic rate for 6 hours. We recruited 11 healthy volunteers, 4 male, median age 23 (IQR 21–25), who completed one-day laboratory studies measuring core body temperature via telemetry and metabolic rate via indirect calorimetry. Participants consumed an oral loading bolus of dexmedetomidine (2 μg/kg) followed by a six-hour SQI of dexmedetomidine (1 μg/kg/hr). Surface cooling pads were placed on the backs of 7 participants to promote heat loss. We collected vital signs continuously and monitored participants until they could be safely discharged. Energy expenditure (EE; kcals per day) dropped from baseline regardless of surface cooling. With surface cooling, median temperature decreased from 36.9°C (IQR 36.7–37.0°C) at baseline to 35.4°C (IQR 35.3–35.5°C) at 6 hours. Sublingual loading dose followed by 6-hour SQI of dexmedetomidine safely and effectively reduces metabolic rate. Future studies should be evaluating the effectiveness of SQI dexmedetomidine without a sublingual loading bolus, evaluating novel administration methods, and determining if tolerance develops with long-term use.

## Introduction

Reducing metabolic rate and temperature is an adaptation to periods of limited resources and environmental stressors for many animals [1]. This metabolic reduction is often characterized by a reduction in activity, heart rate (HR), core body temperature and oxygen consumption. The duration and extent of this metabolic reduction varies between species [2]. Some animals undergo a shorter period of significant metabolic reduction that lasts days to weeks [3]. Others undergo a longer period of metabolic reduction lasting weeks to months [4]. While humans do not naturally experience hibernation, or subsequent metabolic reduction, artificial induction of metabolic reduction may be potentially beneficial. In medical treatment, lower metabolic rate protects the heart and brain during myocardial infarction or stroke while awaiting reperfusion [5,6]. In extreme environments (spaceflight, austere terrestrial environments, e.g., Arctic) or during emergent situations where consumable resources are limited, reduced metabolism could prolong survival until conditions improve, or until rescue. However, only short-term and highly invasive methods for metabolic reduction have been explored in humans. For example, the combination of anesthesia and hypothermia protects the brain and other organs during surgical procedures during times of restricted blood flow [7]. Hypothermia has also been used to treat cerebral ischemia, but it is unclear whether this effect is related to temperature effects on metabolic rate [8]. Practical methods to reduce metabolism and temperature in non-anesthetized humans would allow exploration of these applications and questions.

Decreasing ambient temperature or forced lowering of body temperature in non-hibernating euthermic animals, including humans, usually leads to an increase in metabolic rate as part of the homeostatic response to regulate core body temperature [9]. This cold-induced thermogenesis (CIT) can include shivering and increased energy use in brown adipose tissue [9]. However, in animals during hibernation, metabolic rate decreases both spontaneously and as body temperature decreases, reflecting a suppression of CIT [10,11]. We demonstrated that certain drugs can prevent the increase in metabolic rate with falling temperatures that is characteristic of CIT in humans for several hours, allowing temperature-dependent decreases in metabolic rate [12–15]. Importantly, we identified drugs, such as the alpha-2 adrenergic receptor (A2AR) agonist dexmedetomidine, that can prevent CIT without suppressing breathing or inducing deep anesthesia that would require mechanical life support [12–15]. Dexmedetomidine binds to and activates A2Ars, which are found throughout the central nervous system and mediate pain, sedation and body temperature [16–18]. Within the brain, effects of dexmedetomidine are related to the ventrolateral preoptic nucleus and tuberomammillary nucleus of the hypothalamus, and the locus coeruleus [16]. For example, in healthy humans receiving dexmedetomidine, metabolic rate decreases 5.2% for every 1ºC decrease in core temperature from 37ºC to 33ºC [12]. However, these experiments have not determined whether safe and sustained metabolic reduction and prevention CIT is practical in non-anesthetized humans.

We explored a drug-induced reduction of metabolic rate and CIT using a sustained infusion of dexmedetomidine in healthy volunteers. We used subcutaneous infusion of dexmedetomidine (SQI) as a less invasive strategy, since prolonged intravenous drugs would not be practical in some settings. Specifically, we tested whether dexmedetomidine infusion with and without surface cooling can decrease metabolic rate and prevent CIT for 6 hours.

## Methods

### Study design

The University of Pittsburgh Human Research Protection Office approved this protocol. We enrolled a total of 13 participants between March 14 and June 29 of 2023 (Table 1). We assessed physiologic measures 1 hour prior to and 6 hours after a sublingual loading dose of 2 µg/kg of dexmedetomidine followed by a 1 µg/kg/hr dexmedetomidine SQI. The 1-hour period prior to drug administration served as a within-participant control to approximate resting metabolic rate. Participants had surface cooling with cold water circulating via gel-adhesive pads placed on their back. In four participants, we omitted surface cooling to observe the effects of drug alone.

**Table 1:** Participant characteristics.

	Cohort (N=11)	With Cooling (n=7)	Without Cooling (n=4)
**Age**	23 (21-25)	23 (21-26)	23 (21-24)
**Female Sex (%)**	7 (64%)	4 (57%)	3 (75%)
**Race**			
Asian	2 (18%)	1 (13%)	1 (25%)
Black/ African American	3 (27%)	2 (28%)	1 (25%)
White	7 (64%)	5 (71%)	2 (50%)
**Ethnicity** Non-Hispanic/ LatinX	11 (100%)	7 (100%)	4 (100%)
**Height (cm)**	173 (168-175)	173 (168-175)	171 (169-174)
**Weight (kg)**	67.3 (66.7-81.1)	76.3 (66.7-84.6)	67 (64.2-74.2)
**BMI (kg/m**^**2**^)	23.9 (22.4-26.4)	25.6 (22.4-29.2)	23.1 (21.8-25.2)
**Predicted BMR (kcal/d)**	1460 (1445-1812)	1491 (1445-1812)	1453 (1424-1636)
**Percent Body Fat**	18.5 (14.7-21.8)	18.1 (14.7-21.8)	20.15 (16.6-22.3)
**VO**_**2**_ **Max (ml/kg/min)**	37 (32.9-38.5)	37 (32.9-40.1)	36.5 (34.5-37.8)
**Grip Strength (kg)**	70 (68-92)	70 (68-92)	70.5 (65-88.5)

Data are represented as median (interquartile range), or percentages.

### Setting

To reduce circadian variation between experiments, we administered drug between 10am and 11am. Ambient laboratory environment included constant light (350–400 Lux) and noise levels (~55dB), median temperature 22°C (IQR 21°C-23°C), and humidity 36% (IQR 34–42%). Throughout the protocol, participants lay supine on a stretcher with their head elevated and supported with pillows to their comfort. To create a comfortable environment, participants dressed in athletic shorts and tops for all activities, removed their shoes while resting on the stretcher, and a light blanket was provided to each participant.

### Participants

We recruited healthy volunteers with informational flyers in and around the University of Pittsburgh campus and advertised our study on the University of Pittsburgh’s human research website (Pitt+Me). Participants provided written, informed consent prior to engaging in any research activities. Participants were enrolled between March 14 and June 29 of 2023.

### Screening

We screened all volunteers prior to study engagement to ensure their safe participation. Participants were required to be between 18–55 years of age, non-smokers (including vaping), non-tobacco users, without sleep disorders or past medical history of a chronic health condition that would interfere with the protocol (diabetes, chronic lung disease, known cardiac disease, thyroid disease). We excluded individuals if they had an allergy to dexmedetomidine, were taking medications that interact with dexmedetomidine, reported habitual use of sleep aids, had a prior abdominal surgery that would prevent use of ingested thermometer capsule, or if they had claustrophobia that would prevent them from resting comfortably under a translucent hood during metabolic assessment.

Participants completed the Physical Activity Readiness Questionnaire (PARQ+) to confirm healthy status and readiness for exercise participation [19]. On physical examination we confirmed that physical characteristics were within the following target ranges: mass (>55 kg), body mass index (BMI) (18.5–30 kg/m^2^), resting heart rate (HR) (50–100 beats per minute [bpm]), resting systolic blood pressure (100–150 mmHg), and diastolic blood pressure (60–90 mmHg). We performed a 12-lead electrocardiogram and excluded individuals with resting dysrhythmias or Atrio-Ventricular conduction delays. We used the Epworth Sleepiness Scale (ESS) to screen participants for excessive daytime sleepiness, defined as ESS ≥ 11 [20].

To assess fitness status, we assessed both muscular strength and maximal oxygen consumption (VO_2_ Max). To obtain VO_2_ Max, participants completed the modified Bruce Treadmill Protocol in which treadmill incline and speed increases every three minutes until volitional maximal exertion, or RER>1.1 [21]. We quantified VO_2_ via open circuit spirometry where a participant dons a face mask and inspired and expired gases are analyzed (Parvo-medics Inc., Parvo TrueOne 2400, Sandy, UT, USA). We used grip strength as a surrogate measure of overall muscular strength [21], using a hand dynamometer (JAMAR, Chicago IL, USA) with the participant in the standing position. Participants held the handgrip dynamometer in line with the forearm at the level of the thigh, away from the body. Grip strength was calculated as the highest of two readings for each hand (rounded to the nearest kg) added together [21]. We excluded volunteers if their VO_2_ Max was <1 SD below, or >2 SD above the mean for their age and sex, or if their maximal grip strength was not within ± 2 SD of the mean for their age and sex.

### Metabolic assessment

We used participant height, mass, sex, and age to predict basal metabolic rate (BMR) [22]. Prior to each experiment, we calibrated the metabolic cart with standardized gas concentrations (AirGas USA, Plumsteadville, PA) and flow calibration standards according to the manufacturer’s system requirements. We performed indirect calorimetry by measuring CO_2_ excretion and O_2_ consumption using the ventilated-hood (canopy) technique (Parvo-medics Inc., Parvo TrueOne 2400, Sandy, UT, USA). This method required participants to rest quietly in the supine position under a clear canopy. Inlets and outlets on the canopy hood allowed a fan on the metabolic cart to pull air through the canopy and into the metabolic cart’s gas mixing chamber at a constant rate (~15–25 l/min). The system continuously monitors gas concentrations within the mixing chamber and in ambient air, to inspired and expired gases, and a computer records mean values every minute. The metabolic cart is pre-programmed to use VO_2_, and VCO_2_, and the Weir formulas to derive respiratory exchange ratio (RER), and energy expenditure (EE, measured in kCal) [23].


$$EnergyExpenditure=\left(3.941xVO_{2}+1.106xVCO_{2}\right)/\left(1+0.082x0.125\right)$$

$$RER=VCO_{2}/VO_{2}$$

$$VO_{2}=\left({\left[O_{2}\right]}_{inspired}-{\left[O_{2}\right]}_{expired}\right)xflowrate$$

$$VCO_{2}=\left({\left[CO_{2}\right]}_{inspired}-{\left[CO_{2}\right]}_{expired}\right)xflowrate$$

As a baseline assessment, we measured resting metabolic rate for a minimum of one-hour prior to drug administration.

### Temperature manipulation

For participants undergoing hypothermia, we placed two water-circulating, gel-adhesive pads (Arctic Sun Universal Pad, Arctic Sun, Franklin Lakes, NJ, USA) for surface cooling on the back of each participant depending on the size of their torso. Each pad has a surface area of 0.17m^2^, which allowed for 8–15% of total body surface area to be covered by the surface cooling pads. We set the water temperature to 15ºC after the drug was administered.

### Physiologic monitoring

We collected continuous measurement of ECG rhythm, finger-clip pulse oximetry, finger-clip pulse plethysmography, and respiration rate (via chest strap placed below the nipple line where maximal chest expansion occurs during inhalation). We calculated heart rate from beat-to-beat intervals from the ECG or finger plethysmography. These continuous measures were collected and converted to digital recordings at a rate of 1000 samples per second using an analog to digital converter (AD Instruments, Sydney, Australia).

We measured blood pressure every 15 minutes via automated sphygmomanometer (Edan Instruments Inc, Shenzhen, PR, China). We measured stroke volume index (SVI) and cardiac index (CI) noninvasively using a bioreactance device (NICOM, Cheetah Medical, Newton Center, MA, USA).

### Temperature measurement

Participants ingested a telemetry thermometer capsule (eCelsius [using research mode], BodyCap, Hèrouville Saint-Claire, France). This capsule relayed deep gastric or enteral temperature to an external receiver every 15 seconds. Software allows for the raw data to be exported in various formats for analysis.

### Shivering assessment

Trained study team members rated participant shivering based on the Bedside Shivering Assessment Scale (BSAS) every 15 minutes. The BSAS ranges from 0–3; with 0, indicating no shivering, and 3, indicating severe uncontrolled shivering of the trunk and all extremities [24].

### Drug administration

We administered dexmedetomidine via a sublingual loading bolus of 2 mcg/kg, followed by a six-hour SQI at a rate of 1mcg/kg/hr. For the sublingual loading dose, we dripped the dexmedetomidine (50 mcg/ml) into the participant’s mouth and instructed them to hold the liquid in their mouth for 60 seconds, after which time they could swallow any remaining liquid. After 60 more seconds, the participant rinsed their mouth with water. The 6-hour infusion started immediately following the sublingual loading dose using a syringe pump (MedFusion 3500 Syringe Pump, Medex Inc. Duluth, Georgia, USA). SQIs ran through a plastic catheter (Medtronic Minimed 6mm or 9mm Quick Set, Northridge, CA, USA) placed aseptically through the skin of the anterior abdomen.

### Plasma drug levels

An intravenous catheter (IV) was inserted into the antecubital vein prior to study initiation both for safety and to assess plasma drug levels. We collected 4 ml of blood in EDTA tubes at baseline, 30, 60, 90, 120, 180, 240, 300, 360, 420, and 480 minutes. We centrifuged samples immediately at a rate of 3000 x g for 20 minutes and aliquot the plasma into cryotubes before storing them in a -40ºC freezer until time of assay.

We developed and validated a LC-MS/MS assay according to FDA Bio-analytical Method Validation Guidance for Industry for the quantification of dexmedetomidine in human plasma [25,26]. We have previously reported this methodology. This assay showed excellent inter- and intra-day linearity, accuracy, and precision [27].

### Study protocol

Participants arrived at the laboratory by 0800, at which point a study team member confirmed the participant had no changes in health since time of screening and a urine human chorionic gonadotropin (HCG) test was performed for all participants capable of becoming pregnant. Once the participant was deemed safe to participate in the study, they ingested a core temperature pill and a trained study team member placed all physiologic instrumentation, including an IV catheter for blood draws and safety measures. We completed a minimum of one-hour of baseline physiologic monitoring and resting metabolic rate assessment prior to drug administration. After baseline assessment, the participant ingested the sublingual loading dose, and we started the SQI. Participants rested quietly or slept throughout the protocol. We continuously recorded physiologic measures and metabolic rate during the six-hour infusion. If participants required use of the toilet, they were assisted by a study team member. Upon completion of the six-hour infusion, we continued to collect metabolic assessments and physiologic parameters until the participant began to arouse from dexmedetomidine administration. Upon arousal, we deescalated instrumentation based on the participant’s level of arousal and discomfort. A study physician discharged the participant once they expressed they were no longer feeling subjective side effects of the drug and their vitals returned to within normal limits. At study completion, the participants were de-instrumented and discharged to the care of another adult. We performed next day follow up via phone call or text message to confirm participants’ continued well-being.

### Statistical analyses

We estimated 8 participants were required to provide 90% power to detect a change in metabolism of at least 18%. The overall goal of the project was to reduce daily energy consumption by 30%, using a combined approach of increasing quiet-rest time to 80% of the day and reducing quiet-resting metabolic rate by 20%. In this study, we examined only the reduction of quiet-resting metabolic rate. We performed all statistical analyses with STATA SE 16 (College Station, TX, USA) and generated graphs using PRISM 7 (GraphPad Software, San Diego, CA, USA).

We collected the raw data for recorded variables and down sampled them into one-minute epochs. For variables that were collected at high sampling rates, such as heart rate (HR), we calculated and recorded the arithmetic mean for each minute. We used all available data for analysis and did not use imputation or interpolation for missing values. We cleaned the data of artifact and false data points resulting from monitor disconnection (e.g., during toilet breaks) using the following guidelines: Heart rate (HR) <30 or >120 (this occurred when the beat-to-beat interval was calculated based on ECG artifact rather than true R-R intervals); Respiratory rate (RR) <3 or >30 (this occurred when the natural rise and fall of the chest was disrupted when a participant was talking); Oxygen saturation <80% (this occurred when the blood pressure cuff inflated above the SpO_2_ finger sensor, or if the finger sensor was disturbed); Core temperature <35 ºC (this level of hypothermia was not attained during this study, lower levels reflected temporary loss of the telemetry signal); Metabolic measurement when fraction of expired CO_2_ <0.5 (this only occurred when the hood was removed from the participant, and could be confirmed by documentation of hood removal). We calculated percent change in energy expenditure (%EE) as the percent change from baseline, and percent difference from predicted basal metabolic rate (BMR) (% Difference).

We report the mean and standard deviation or median and interquartile range of physiologic variables as both change over time and change from baseline. We analyzed changes in physiologic variables with baseline measures averaged into one-minute time epochs, and changes after infusion initiation are reported in 30-minute epochs. We used MANOVA to determine the main effect of time for blood pressure (systolic and diastolic), heart rate, temperature, and metabolic rate. We assessed relationships between change in metabolism and change in temperature; then further explored the association between temperature and metabolism and other physiologic variables with ordinary least squares regression. We defined significance as p ≤ 0.05.

## Results

**Fig 1:**
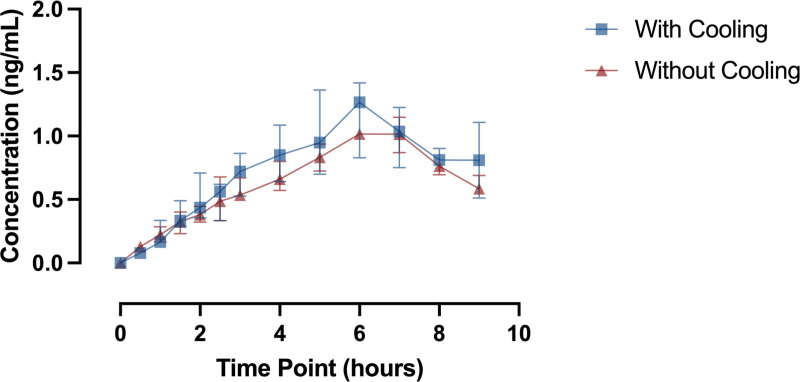
Plasma drug levels by group.

We recruited 13 participants. Leakage in the subcutaneous infusion set prevented drug administration on two participants, which we confirmed by no increase in plasma drug levels after the sublingual loading dose. We excluded data from these two participants leaving 11 participants for analysis (Table 1). Plasma concentration increased steadily for the first 5 hours before plateauing around 0.8–1.2 ng/mL. Dexmedetomidine levels were above 0.8 ng/mL in most participants by 180 minutes (Fig 1).

All participants closed their eyes and remained still during baseline measurements and drug infusion. Predicted BMR (Table 1) correlated well with measured baseline energy expenditure (Table 2) (r=.94, P<0.001). After drug administration, participants became lightly sedated, and we could awaken them by voice. Shivering was minimal both with and without presence of surface cooling (BSAS 0).

**Table 2:** Change in physiologic data over time.

	Group	Baseline	60 min	120 min	180 min	240 min	300 min	360 min
**Temp**	**Cooling**	36.8 (36.7-37.0)	36.6 (36.5-37.0)	36.5 (36.4-36.6)	36.2 (36.1-36.4)	35.9 (35.7-36.1)	35.6 (35.5-35.8)	35.4 (35.3-35.5)
	**No Cooling**	36.7 (36.6-36.9)	36.9 (36.8-37.0)	37.1 (36.7-37.2)	37.2 (36.9-37.3)	37.1 (36.9-37.2)	36.9 (36.8-37.2)	37.0 (36.9-37.2)
**VO**_**2**_ **/kg** **(ml/kg/m)**	**Cooling**	2.93 (2.86-3.31)	2.85 (2.82-3.14)	2.68 (2.34-2.82)	2.62 (2.41-2.70)	2.53 (2.48-2.60)	2.42 (2.30-2.58)	2.39 (2.33-2.53)
	**No Cooling**	3.10 (2.85-3.34)	2.77 (2.58-2.93)	2.71 (2.50-2.86)	2.59 (2.45-2.84)	2.34 (2.13-2.65)	2.22 (2.01-2.62)	2.42 (2.17-2.73)
**VCO**_**2**_ **(ml/min)**	**Cooling**	175 (154.5-246)	149.1 (139-188.1)	140.2 (133.2-170.3)	136.5 (129-164.4)	131.3 (127-155.4)	127 (114-151.3)	123 (113-150)
	**No Cooling**	170 (158.2-223)	143 (135-183)	140 (128.2-181)	144 (124-186)	117.5 (109-213.6)	123 (116-166.2)	120.1 (117-164)
**RER** **(no units)**	**Cooling**	0.81 (0.78-0.83)	0.77 (0.75-0.79)	0.76 (0.75-0.78)	0.75 (0.74-0.78)	0.74 (0.74-0.76)	0.74 (0.73-0.76)	0.74 (0.73-0.75)
	**No Cooling**	0.82 (0.80-0.86)	0.78 (0.86-0.84)	0.78 (0.75-0.83)	0.78 (0.75-0.83)	0.75 (0.73-0.83)	0.77 (0.75-0.80)	0.77 (0.74-0.80)
**EE** **(kcal/d)**	**Cooling**	1493 (1382-1953)	1318 (1236-1624)	1268 (1187-1521)	1220 (1172-1454)	1170 (1157-1413)	1120 (1060-1382)	1125 (1046-1367)
	**No Cooling**	1467 (1346-1815)	1262 (1178-1542)	1231 (1146-1514)	1265 (1124-1551)	1089 (982-1778)	1117 (1047-1433)	1100 (1041-1432)
**% EE** ^a^	**Cooling**	–	-10.1 (-2.5, -18)	-17.3 (-6, -21)	-15.5 (-12, -21.5)	-17 (-13.4, -22.1)	-19.1 (-15, 29)	-19.3 (-18, -30)
	**No Cooling**	–	-14 (-12, -15.3)	-13.3 (-9, -22.2)	-19 (-8, -22)	-27.2 (-15.4, -29)	-22 (-17, - 27.1)	-23 (-18.1, -27)
**% Difference from estimated BMR** ^b^	**Cooling**	-4.4 (-9.5, 7.8)	-12 (-6.8, -15.6)	-15 (-12.3, - 19)	-20 (-16, -21)	-21 (-19, -24)	-23 (-21, -26.3)	-25 (-22, -27.1)
	**No Cooling**	0.81 (11, -6)	-13 (-5.6, - 18)	-14 (-6.3, -21.1)	-13 (-5.3, -21.1)	-25 (-2, -30)	-22 (-12,-28)	-23 (-13, -27.4)

Data are reported as median (interquartile range).

^a^Percent energy expenditure: % EE = (EE _epoch_ – EE _baseline_) * 100/ EE _baseline_

^b^Percent difference from estimated BMR: %Difference EE = (EE _x_ – BMR) * 100/ BMR

**Fig 2:**
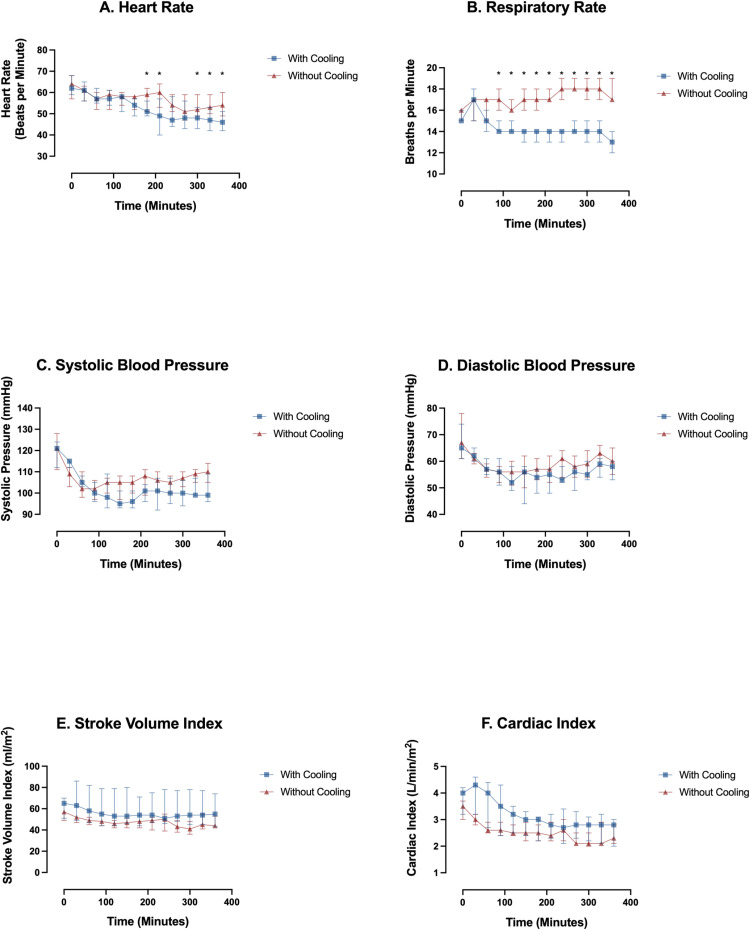
Hemodynamic and respiratory changes over time. Data plotted as median (interquartile range) in 30-minute epochs. * Indicates significant difference in change between groups.

Trends in hemodynamic and respiratory variables are shown in Fig 2. Respiratory rate remained constant throughout the protocol while heart rate, systolic and diastolic blood pressure, cardiac output, and stroke volume index significantly decreased from baseline values within 30–60 minutes after drug induction (P <0.05). However, all stayed within appropriate safety ranges and did not require intervention (Fig 2). Change in systolic and diastolic blood pressure, stroke volume, and cardiac index did not differ by cooling group. Heart rate was significantly different between groups from minutes 150–210 and 270–260 (P <0.05). Respiratory rate was significantly different between groups from minutes 60–360 (P <0.05).

**Fig 3:**
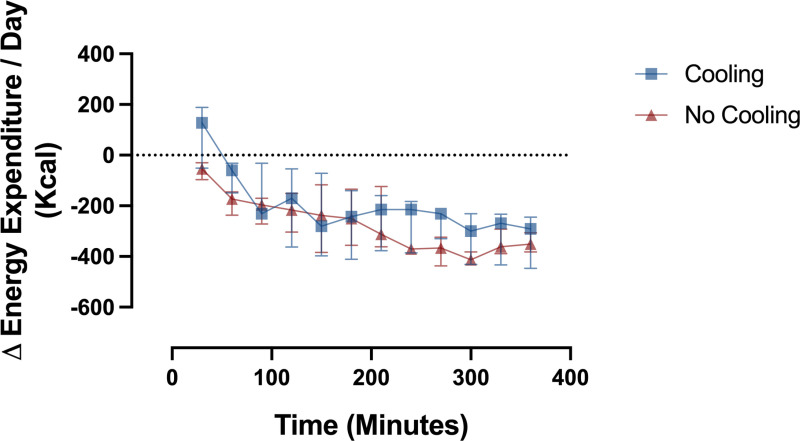
Change in energy expenditure from baseline. Data plotted as median (interquartile range) in 30-minute epochs.

**Fig 4:**
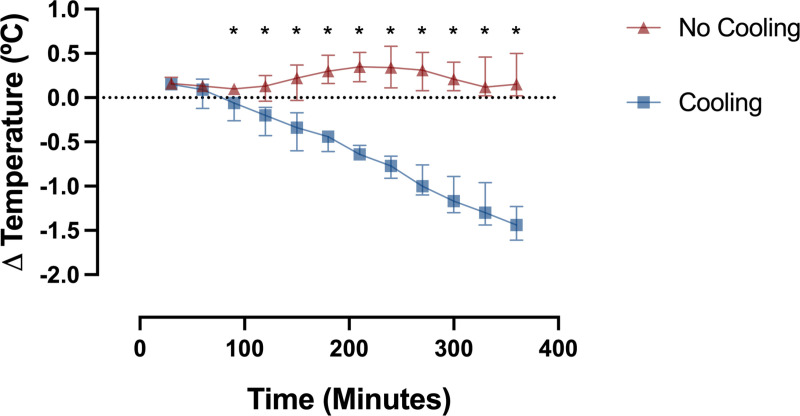
Change in temperature from baseline. Data plotted as median (interquartile range) in 30-minute epochs. * Indicates significant difference in change between groups.

Energy expenditure, expressed as kilocalories per day (kcal/day) decreased relative to baseline within 60 minutes after drug administration and continued to decline over the subsequent 6 hours (P<0.001) (Fig 3). Change in energy expenditure did not significantly differ between cooling and no cooling groups. With surface cooling, temperature declined over the entire 6 hours and differed relative to baseline within 90 minutes of drug administration (P<0.05) (Fig 4). In the absence of surface cooling, temperature remained constant and then increased relative to baseline 150 minutes after drug administration (P<0.05). Change in temperature significantly differed between groups within 90 minutes of drug administration (P<0.05).

**Fig 5:**
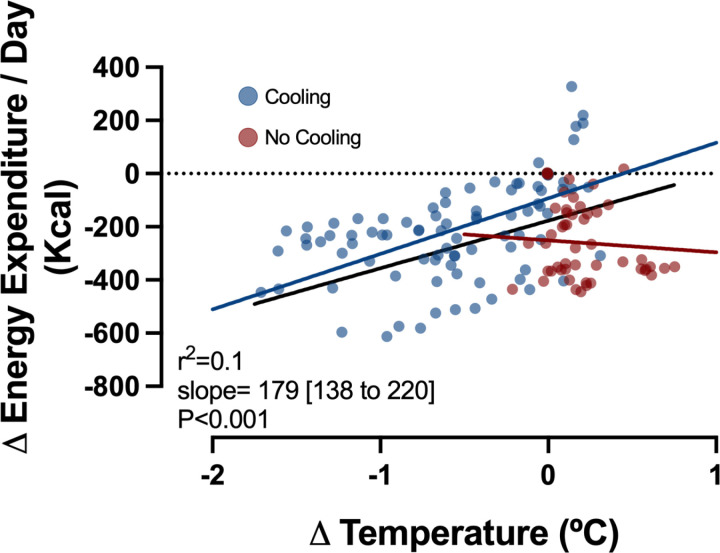
Change in energy expenditure and temperature from baseline. Data plotted as individual median change in energy expenditure by change in temperature.

For all participants (with and without cooling) a decrease in metabolic rate correlated with a decrease in core temperature (slope = 179 (95% CI 138–220), r^2^=0.1). The intercept of the regression of all participants (-177) corresponds to the temperature-independent metabolic reduction from drug alone (Fig 5). For participants who received surface cooling, the decrease in metabolic rate correlated with the decrease in core temperature (slope = 209, (95% CI 175–242), r^2^= 0.30). The intercept of the regression of participants with surface cooling (-93) corresponds to the temperature-independent metabolic reduction from drug alone. In participants who did not receive surface cooling, the decrease in metabolic rate did not strongly correlate with core temperature (slope = -45, (95% CI -234–143, r^2^=0.01). The intercept of the regression of participants without surface cooling (-251) corresponds to temperature-independent metabolic reduction from drug alone.

## Discussion

Continuous SQI of 1.0 µg/kg/hour dexmedetomidine resulted in sustained plasma drug levels of 0.8–1.2 ng/dl, reduced metabolic rate, and, when combined with external cooling, reduced core temperature by 1.4ºC. The positive association between change in temperature and change in energy expenditure in Fig 5, represents inhibition of cold induced thermogenesis: we see energy expenditure decrease with a decrease in temperature. This is in contrast to normative conditions or lower plasma levels of dexmedetomidine, where there is a negative association between change in temperature and change in energy expenditure (e.g., energy expenditure increases with a decrease in core temperature) as the body attempts to maintain homeostasis and resist a decline in core temperature [27]. Decreased metabolic rate and prevention of CIT are physiological changes characteristic of hibernating animals [2,3,11]. Our results suggest that dexmedetomidine effectively replicates this hibernation-like trait in humans by artificially reducing metabolism, and circumventing CIT, which would otherwise be evoked by cooling.

There are two potentially synergistic strategies to reduce metabolic rate. First, is a drug-induced reduction in metabolic rate, which was well demonstrated in this study. Our results indicate that the dexmedetomidine-mediated metabolic reduction preceded the subsequent drop in core temperature. This is consistent with our previous work, and with the order of events typical to naturally-occurring hibernation [27]. However, it differs from the foundation of clinical hypothermia, which works under the assumption that therapeutic hypothermia works due to a temperature-dependent reduction in metabolism [12,13,28,29]. Core body temperature is determined by the balance between heat production and heat loss. Thus, a reduction of metabolic rate with a reduction in heat loss, or with augmented heat loss caused by external cooling, will result in a decrease in core body temperature. Because dexmedetomidine also inhibits shivering and cold-induced thermogenesis, this drop in core body temperature is both tolerated and sustained throughout the 6-hour infusion. This tolerance to reduced core temperature may in part be due to the drug’s vasoconstrictive properties, and its ability to reduce heat exchange at the skin level, thus lowering the shivering threshold [30–32]. The surface-level vasoconstriction may reduce the discomfort or thermal sensation on the skin enough for individuals to better tolerate heat loss. Previous studies have already demonstrated that shivering can be suppressed during cooling with the use of intravenous drugs, including dexmedetomidine, and that shivering will increase metabolic rate [12–15,31–33]. Second, is the reduction in metabolic rate from lowering core body temperature. Cerebral metabolic rate declines about 6% per 1ºC decrease in core body temperature in anesthetized, non-shivering adults [8], in a prior study, we observed a reduction of VO2 of 5.6% per 1ºC decrease in core body temperature in spontaneously breathing, sedated adults [12]. More research needs to be done to further demonstrate this strategy. By directly reducing metabolic rate and allowing core body temperature to decline, drugs like dexmedetomidine could take advantage of both strategies to deliver greater reductions in metabolic rate.

Reduction of energy expenditure, VO2 and VCO2 were similar with and without external cooling, despite the very different temperatures between groups (Table 2). The laboratory climate, clothing participants wore, and light blanket provided for comfort, successfully created neutral thermal conditions, as evidenced by the strong correlation between measured baseline energy expenditure and predicted BMR. These observations suggests that the majority of reduction in metabolic rate resulted from dexmedetomidine and not from temperature change. In addition, most of the reduction in metabolic rate occurred by 60–90 minutes after start of drug (Fig 3), but the temperature continued to decline in participants with external cooling for hours after the decrease in metabolic rate (Fig 4). The best estimate of temperature-independent drug-induced reduction of metabolic rate comes from the regression line in the plot of change in energy expenditure versus change in temperature for all participants (Fig 5), which has a positive slope of 179, and an intercept of -177 kcal/day at ∆T=0ºC.

Changes in other physiological measures were well tolerated. Heart rate, blood pressure, SVI and CI declined after the start of dexmedetomidine, but remained stable for the hours after the initial decline (Fig 2). This is consistent with prior reports of hemodynamic changes with plasma levels of dexmedetomidine <1.9 ng/ml, which decrease circulating catecholamine levels [34]. Respiratory rate did not change by any clinically significant amount, which is consistent with the minimal respiratory depression associated with dexmedetomidine [35,36]. However, the respiratory rates declined more in the participants with external cooling than in those with no cooling (Fig 2). It is possible there is an interaction between lower core temperature and dexmedetomidine that alters the respiratory drive. Because VCO2 and SpO2 did not differ between participants based on cooling, there is no evidence that the difference in respiratory rate was driven by altered CO2-drive or hypoxemia. However, we did not measure tidal volume or end-tidal CO2, we cannot determine if total ventilation declined based solely on respiratory rate. Understanding any drug-temperature interaction on respiratory mechanics will require further study.

Few studies have been done to investigate drug-induced and temperature-induced reductions of metabolism in healthy individuals. To our knowledge, this is the first study to use an oral-loading bolus, followed by a SQI of dexmedetomidine to initiate metabolic reduction. While our study did not demonstrate a difference in metabolic reduction with the addition of cooling, a study by Hostler and colleagues investigating the impacts of hypothermic and thermal neutral conditions on metabolism found a greater reduction in metabolism occurred in hypothermic environmental conditions. While the drug administration method, and study duration differed, these conflicting results further emphasize the need to better understand the relationship between metabolism and temperature [37].

One of our goals was to develop drug regimens for reduction of metabolic rate that could be self-administered. Topical or ingested drugs would be ideal, but we have not yet identified an effective agent with desirable pharmacokinetic profile [27]. The A2AR agonist clonidine may produce similar metabolic effects and is available in a topical patch, but it has a very long half-life [38]. Dexmedetomidine is a small molecule for which development of a similar transcutaneous delivery system should be possible. While the SQI used in this study is more invasive than oral or transcutaneous routes, this method has extensive data showing that it is well tolerated for long-term self-administration of insulin by persons with diabetes [39]. Future work can optimize the delivery system, but the present approach demonstrates proof of concept for self-administered metabolic reduction drugs. Future studies can explore whether participants achieve greater reductions of metabolic rate at lower temperatures during drug-induced and temperature-induced metabolic reduction. Evaluation of SQI dexmedetomidine without an oral loading bolus and novel drug administration methods should also be tested. Nutritional guidelines to reduce thermal effects of food may also have additive metabolic effects when paired with drug and temperature-induced. metabolic reduction. It is also unknown if tolerance is developed with long-term use. Safety and tolerance in extreme environments (spaceflight and austere terrestrial environments, etc.) will need to be explored.

### Limitations

We only studied four participants without external cooling. However, the data from these participants was comparable to the cooled participants and the data were sufficient for us to separate temperature-dependent and temperature-independent effects on metabolic rate. The sample size was both small and of narrow age range. All study protocols began in the morning, which prevents us from evaluating the influence of natural changes in temperature and metabolism that occur due to circadian rhythms on the effectiveness of the protocol.

## Conclusion

An oral loading dose followed by a 6-hour SQI of dexmedetomidine safely and effectively reduces metabolic rate. Drug-induced metabolic reduction and lowering of core body temperature are two potentially synergistic strategies to reduce metabolic rate.
